# Validation of the care providers version of the Rainbow Model of Integrated Care-measurement tool in Chinese primary care systems

**DOI:** 10.1186/s12913-020-05562-2

**Published:** 2020-08-08

**Authors:** Yixiang Huang, Paiyi Zhu, Lijin Chen, Xin Wang, Pim Valentijn

**Affiliations:** 1 School of Public Health, Health Development Research Center, Sun Yat-Sen University, Guangzhou, 510080 China; 2 Department of Health Services Research, Care and Public Health Research Institute (CAPHRI), Faculty of Health, Medicine and Life Sciences, Maastricht University, Maastricht, The Netherlands; 3 Integrated Care Evaluation, Essenburgh, Hierden, The Netherlands

**Keywords:** Integrated primary care, Measurement tool, Community health professionals

## Abstract

**Background:**

The original Rainbow Model of Integrated Care Measurement Tool (RMIC-MT) is based on the Rainbow Model of Integrated Care (RMIC), which provides a comprehensive theoretical framework for integrated care. To translate and adapt the original care provider version of the RMIC-MT and evaluate its psychometric properties by a pilot study in Chinese primary care systems.

**Methods:**

The translation and adaptation process were performed in four steps, forward and back-translation, experts review and pre-testing. We conducted a cross-sectional study with 1610 community care professionals in all 79 community health stations in the Nanshan district. We analyzed the distribution of responses to each item to study the psychometric sensitivity. Exploratory factor analysis with principal axis extraction method and promax rotation was used to assess the construct validity. Cronbach’s alpha was utilized to ascertain the internal consistency reliability. Lastly, confirmation factor analysis was used to evaluate the exploratory factor analysis model fit.

**Results:**

During the translation and adaptation process, all 48 items were retained with some detailed modifications. No item was found to have psychometric sensitivity problems. Six factors (person- & community-centeredness, care integration, professional integration, organizational integration, cultural competence and technical competence) with 45 items were determined by exploratory factor analysis, accounting for 61.46% of the total variance. A standard Cronbach’s alpha of 0.940 and significant correlation among all items in the scale (> 0.4) showed good internal consistency reliability of the tool. And, the model passed the majority of goodness-to-fit test by confirmation factor analysis.

**Conclusions:**

The results showed initial satisfactory psychometric properties for the validation of the Chinese RMIC-MT provider version. Its application in China will promote the development of people-centered integrated primary care. However, further psychometric testing is needed in multiple primary care settings with both public and private community institutes.

## Background

Health systems in some low- and middle-income countries, as well as in most high-income countries, face the challenges of aging populations and rising chronic disease prevalence [1, 2]. These growing challenges call for more integrated approaches instead of the current single-disease and acute-care-focused health care systems. The World Health Organization (WHO) acknowledges integrated care in its vision and global strategy for health care delivery [3]. There has been a proliferation of integrated care initiatives in many different countries and settings [4]. Furthermore, some researches have demonstrated that integrated care contributes to improving the 3 components of Triple Aim, improved population health, improved individual experiences of care and reducing costs of per capita care [5–7]. While the aims are promising, integrated care remains a complex health intervention involving multiple levels of organizations with multiple care providers, multiple interventions, and multiple contextual factors that can influence processes and outcomes of care delivery [8, 9]. Measuring care providers’ experience and behavior in a consistent way is critical for evaluation of the implementation of integration interventions and advancement of the success of health care integration [10], since multiple care providers are the final driver of providing integrated care.

Several instruments have been developed for the assessment of integrated care [11–13]. For example, Suter et al., identified 114 instruments, over half of which were self-reporting questionnaires that measured care coordination and patient and family involvement, by a knowledge synthesis of indicators and measurement tools for health system integration [12]. Bautista et al. conducted a comprehensive systematic review based on the Rainbow Model of Integrated Care (RMIC) [11]. They also confirmed that patient-focused dimensions, such as patient-centered care and care integration, were the most common measuring constructs in majority of studies. Furthermore, they found that less than half of the instrument validation studies were of good quality for the measurement of properties. Only a few research reports assessed integrated care from the perspective of healthcare professionals [14–16]. Stephenson et al., performed a rapid review of all quantitative surveys and qualitative research studies to assess healthcare professionals’ experiences with integrated care [14]. They reported that the common dimensions of existing quantitative surveys were communication, agreement on clear roles and responsibilities, facilities, information systems, coordination of care and access, which missed some deeper aspects affecting teamwork such as trust between and among providers, and management in the institutes [17]. In summary, there are three deficiencies of the existing instruments. First, it remains an area for potential validated instruments to assess integrated care from the perspective of multiple care providers. Second, organizational and system integration dimensions and normative enabling factors are not addressed in current instruments. Third, weak psychometric validity and reliability measurement of current instruments.

The RMIC was developed as a result of literature reviews in primary care settings, and was validated by a series of Delphi panels and a panel of international experts [18]. Subsequently, the Triple Aim framework was synthesized into the model by Delphi studies with an interdisciplinary panel of experts [19]. The RMIC distinguishes four integrated care dimensions (clinical integration, professional integration, organizational integration, system integration), two enablers (functional integration, and normative integration) at micro- meso- and macro-levels, two guiding principles of integration (person-focused care and population-based care), and the three interrelated outcome dimensions (population health, experience of care and cost). The RMIC provides a comprehensive framework for integrated care from the perspectives of both patients and care providers, combining the implementation and outcome measurement of integrated care. Based on the RMIC and more than 300 integrated care instruments for healthcare providers and patients, the RMIC-MT (measurement tool) was developed. In addition to micro-level measurements in previous quantitative surveys, RMIC-MT care provider version adopted items at meso- and macro-levels, such as regional healthcare context and performance management, which may more deeply influence the implementation of healthcare integration [20].

The RMIC-MT was developed based on a literature review and two international Delphi studies. The preliminary version of the RMIC-MT provider version has been tested in the Netherlands, Australia, and Singapore [21–24]. These studies indicated that further research was needed to improve the psychometric properties of the professional, organizational, system, functional and population-based scales. Therefore, in international validation across 19 countries has been conducted in 2017 [25]. The results of this study indicated that the internal consistency, reliability, and construct validity of the RMIC-MT (36 items, 9 subscales) provider version was good. These results showed that the RMIC-MT provider version is a valuable psychometric tool for evaluating integrated care initiatives in various countries. This study aimed to validate the Chinese RMIC-MT care provider version in the context of the implementation of integrated primary care [18]. Application of this tool could promote regular evaluation of integrated care and further implementation of integrated health systems in China.

## Methods

**Fig. 1:**
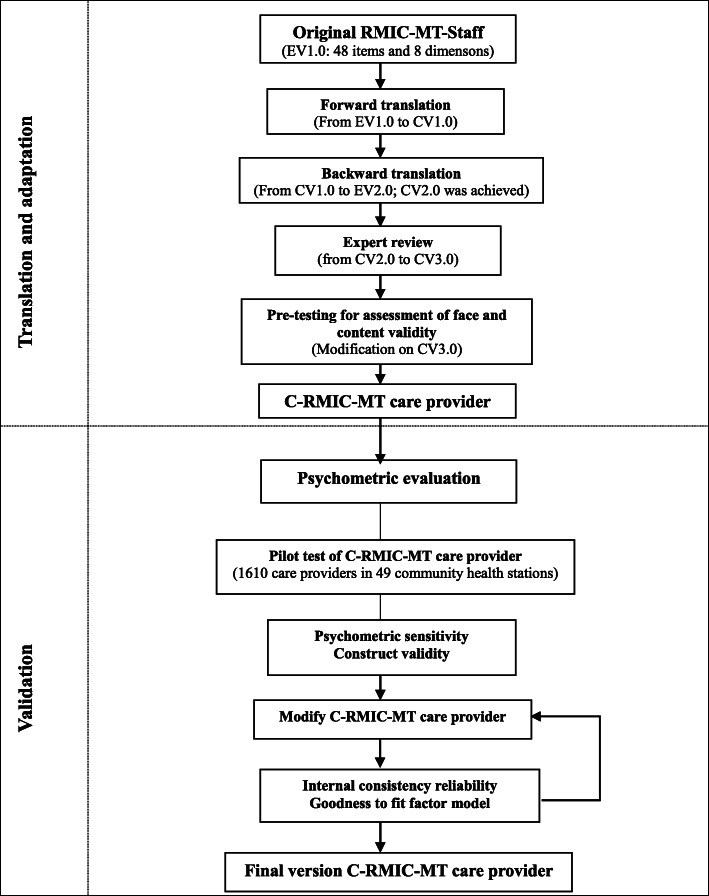
Study design

The English RMIC-MT care provider version as used in the international validation study was tested for its validation in Chinese primary health systems [25, 26]. The validation was conducted in two phases, the translation and adaptation process, and the assessment of psychometric properties (Fig. 1).

### The structure and scoring of the original RMIC-MT care provider version

Based on the dimensions in the Rainbow Model, The original RMIC-MT care provider version consists of 48 items grouped into eight dimensions: (1) Person-centeredness (5 items), (2) Community-centeredness (4 items), (3) Clinical Integration (7 items), (4) Professional Integration (7 items), (5) Organizational Integration (4 items), (6) System Integration (3 items), (7) Technical competence (10 items), (8) Cultural competence (8 items). Items in dimensions 1, 2, 5, 6 are answered on a 5-point Likert scale ranging from “strongly disagree” to “strongly agree”, with rating scores from 1 to 5. Items in the dimensions 3, 4, 7, and 8 are answered on a 5-point Likert scale ranging from “never” to “always”, with rating scores from 1 to 5. There are five reverse questions (items 17, 18, 19, 22, 23) in the dimension “Professional Integration”, which are also answered on a 5-point Likert scale ranging from “never” to “always” with rating scores from 5 to 1. The total RMIC-MT care provider score is computed by summing the scores on each item, with a maximum score of 240 points. Providers were also asked to rate the overall perceived ability to coordinate care internally and externally on a -point scale ranging from very poor (1) to excellent (10). The two questions were how did you rate coordination among CCPs inside and outside your CHS. In addition, the adaptive reserve of the CHS was assessed using the resource and culture subscale on a five-point Likert scale derived from the work of Helfrich et al. [27].

### The translation and adaptation process

According to Harkness and Streiner [28, 29], we utilized a four-step systematic approach for the translation and adaptation process: forward translation, back-translation, expert reviews, and pre-testing of content validity.

#### Forward translation

Two postgraduate students with Chinese as their first language, and majoring in Health Economics and Policy (HEP), independently translated the RMIC-MT-Staff into Chinese independently. By comparing the two forward translated versions, the author (X.W.) reviewed the differences and discussed them with the two translators until they all agreed on a reconciled Chinese version 1.0 (CV1.0).

#### Back-translation

The CV1.0 was translated back into English by two PhD candidates majoring in HEP, who had never read the original English version. Both translators are bilingual translators with English as their first language. The back-translated and original English versions were compared and discussed with the author (X.W.) and the two translators with the aim of reaching satisfactory equivalence between EV2.0 and EV1.0. CV2.0 was agreed based on modification after the discussion.

#### Expert review

Semi-structured interviews were conducted independently with four Chinese experts working on integrated care or primary care to obtain their reflections on the suitability of the CV2.0 of the RMIC-MT care provider version for use and the reasons underpinning the responses. The CV3.0 was obtained based on the experts’ reflections.

#### Pre-testing for assessment of face and content validity

Pre-testing was conducted with two policy makers and 12 community care professionals (CCPs). Through a face-to-face interview, each participant was asked to review each item on the CV3.0, and comment on wording, the length of the survey, and the relevance of the items in the Chinese setting. The relevant items were rated as 1 and the unnecessary items rated as 0, and a content validation index was calculated to test content validity of the CV3.0. After the pre-testing phase, two researchers (X.W. and P.V.) discussed the final modification C-RMIC-MT care provider version.

### The pilot test of the C-RMIC-MT care provider version

#### Sampling and data collection

After the translation and adaptation process, the final version was used in a pilot study that launched the establishment of a medical consortium. In April 2017, the General Office of the State Council issued a guideline for constructing medical consortia [30], making them a main means for achieving people-centered integrated care. As a response to the new policy, policymakers in Nanshan began construction of a medical consortium with the purpose of strengthening cooperation between hospital care and community care, especially enhancing community care. There are 79 community health stations (CHSs) in the Nanshan district under the unified management of the medical consortium. In these 79 stations are 1784 working CCPs, including general practitioners (GP), nurses, public health physicians and the others. CCPs of the 79 CHSs, who were at work in the last three months and able to complete online questionnaire independently, were invited to participant in the survey. Data were collected by using an online data collection tool “SO JUMP [31]”. In July 2018, links to the online questionnaire were sent to all potential participants’ mailboxes through an automatic office system. Once the online questionnaire was completed, it was re-submitted to “SO JUMP”. After logging in, authors could immediately download all original data.

### Data analysis

Scales with more than 30% missing data were excluded from the analysis. After the cleaned data were entered, psychometric sensitivity, construct validity, concurrent validity and internal consistency were analyzed for the assessment of psychometric properties of the C-RMIC-MT care provider version by SPSS 20 and Amos.

#### Psychometric sensitivity

Distribution of responses to each item was analyzed for the study of psychometric sensitivity. Items with skewness value > 3 and kurtosis value > 7 [32], or items with a floor or ceiling effects of > 75% of respondents, were considered for deleting as sensitivity problems [33].

#### Validity

The Kaiser-Meyer-Olkin (KMO) measure of sampling adequacy and Bartlett’s test of sphericity were used first to determine if exploratory factor analysis (EFA) could be conducted [34, 35]. With a KMO value over 0.8 and a significant Bartlett’s test, EFA analysis could be conducted to assess construct validity [36]. EFA with principal axis factoring extraction method and promax rotation were used to assess the underlying structure. EFA and promax rotation in this study followed the description by Brown [37, 38]. The number of factors was determined by consideration of the eigenvalue (> 1), scree plot, and interpretability of the factor. More importantly, the factors retained had to be guided theoretically. Names were given for each identified factor based on the dimensions of the RMIC. Items that cross-loaded on more than one factors were placed with the factor that was most closely related conceptually. Items with poor factor loading (< 0.4) were removed from the questionnaires.

Construct validity was assessed by the calculation of Pearson’s correlations between the scale scores and two overall perceived coordination questions within the questionnaires. Moderately positive associations (≥ 0.4) between the score of the scale and these correlations would indicate good construct validity [39]. The following hypotheses were tested: 1) care providers who indicate a better care coordination ability are more satisfied with the internal adaptive reserve and external care coordination ability; and 2) each subscale aims to measure the CHS coordinated care ability and are therefore positively and significantly correlated with other subscales. *P*-value < 0.05 were considered statistically significant.

#### Internal consistency reliability

Based on potential modification in the above two phases, internal consistency reliability was assessed by items-total correlations and Cronbach’s alpha. Items-total correlations coefficients between items within a scale should be ≥ 0.4 [40]. If Cronbach’s alpha ranged between 0.70 and 0.95, the scale was considered reliable for use in the sample population [41]. Moreover, Pearson correlation coefficients were calculated to assess whether each item was correlated the highest on an assigned subscale by correlation of items with the subscale means. If no correlation was achieved, the item would be eliminated [42].

#### Goodness to fit factor model

Confirmation factor analysis was used to evaluate the explorative factor analysis model fit by using the standard fit indices: root-mean-square error of approximation RMSEA (≤0.06), standardized root-mean-square residual (SRMR) (≤0.08), comparative fit index (CFI) (≥0.80), Tucker-Lewis index (TLI) (≥0.80).

## Results

### The translation and adaptation process

The back-translation and the original version were basically identical. Only a few words were modified because it was difficult to find words in Chinese conveying the same meaning. For example, the word “coordinate” was replaced by “borrow or rent” in item 26, and by “expert consultation” in item 27. ‘Coordinate’ is a word not commonly used in daily life and has general meaning in Chinese. The distinction between “discipline” and “professional” is not familiar in Chinese. During the expert review, four experts gave some suggestions fitting Chinese primary care systems. For example, they suggested taking GPs and public health physicians as members of a multidisciplinary team, rather than psychologist and dietitian in item 15 of the original version. They suggested deleting “transfer” in item 11, since it is not the health institutes’ responsibility to transfer patients except in an emergency. Additionally, the experts suggested reordering the ten dimensions in the original version, to make it more easily understood for completion by Chinese-speaking individuals. In the pre-testing, the content validity analysis showed a content validation index of 0.84, which is slightly higher than the recommended level of 0.80. Some of the 14 participants in the pre-testing replied that they had difficulty in imagining the specific scenario described in the question, so they suggested more detailed examples, such as a specific action or regulation. After discussions with the developer (P.V.) of the original RMIC-MT care providers version, the author (X.W.) kept all 48 items and made some detailed modifications (Table 1).

**Table 1:** Detailed modifications in the translation and adaptation process

No.	Original version	Modification	Reasons
1	Change orders of the ten dimensions	Person-centeredness (items 1–5) Community-centeredness (items 6–9) Clinical Integration (items 10–16) Organizational integration (items 17–20) System integration (items 21–23) Professional integration (items 24–30) Technical competence (items 31–40) Cultural competence (items 41–48)	Making the questions more easily asked, understood, and completed by Chinese.
2	Item 6: Within this clinic, it is important to work with community-based service organizations to improve delivery of care.	insert “e.g. neighborhood committee” at the end of “community-based service organizations”.	Cite an example.
3	Item 10: Within this clinic, written plans and schedules are used to coordinate care for patients.	Insert “(e.g. patient referrals and expert consultation)” at the end of the question.	Cite examples of coordinating care for patients.
4	Item 11: Within this clinic, written plans and schedules are used for patient referrals, transfers, and follow-up with care providers outside the clinic (e.g. hospital, cardiologist, etc.).	Delete “transfers”.	It is not the health institutes’ responsibility for transferring patients except in an emergency.
5	Item 15: Within this clinic, there is a multidisciplinary team (e.g. psychologist, dietitian etc.).	Delete “psychologist, dietitian” and insert “general practitioners and public health physicians”.	General practitioners and public health physicians are required members of multidisciplinary teams of CHS in China.
6	Item 25: This clinic coordinates with other organizations in the region to eliminate unnecessary duplication of administrative services.	Insert “(e.g. repeated registration)” at the end of the question.	Cite an example.
7	Item 26: This clinic coordinates the use of its technology and equipment with other organizations in the region to provide better care for patients.	Insert “(e.g. borrow or rent)” at the end of “coordinates”.	Cite an example.
8	Item 27: This clinic coordinates the use of its staff/personnel with other organizations in the region to provide better care for patients.	Insert “(e.g. expert consultation)” at the end of “coordinates”.	Cite an example.
9	Items 28/29/30	Insert one most recent specific health regulation for each item.	Cite an example.

### Psychometric results

Among all 1784 CCPs in 79 CHSs of Nanshan district, 1610 (90.4%) replied to our invitation. Table 2 summarizes the characteristics of the participants.

**Table 2:** Characteristics of participants

Characteristics	Number (***n*** = 1610)	Percent
**Gender**		
Male	448	27.83%
Female	1162	72.17%
**Age (years)**		
< 30	441	27.39%
30–49	1098	68.20%
≥ 50	71	4.41%
**Marital status**		
Married	1271	78.94%
Others	339	21.06%
**Level of education**		
Junior technical college	92	5.71%
Senior technical college	532	33.04%
Undergraduate and graduate-university	986	61.24%
**Years of work experience**		
< 5	337	20.93%
5–10	327	20.31%
< 10	946	58.76%
**Position**		
GP	517	32.11%
Specialist	61	3.79%
Public health physician	55	3.42%
Traditional Chinese medicine physician	87	5.40%
Nurse	676	41.99%
Pharmacist	76	4.72%
Laboratory workers	58	3.60%
Chemist	1	0.06%
Practitioner of traditional Chinese medicine	16	0.99%
Health manager	10	0.62%
Administrative staff	53	3.29%
**Income (¥/month)**		
< 3000	112	6.96%
3000-4999	575	35.71%
5000-7999	642	39.88%
8000-11,999	234	14.53%
≥ 12,000	42	2.61%

#### Psychometric sensitivity

Distribution analysis of responses to each item showed that there was no item with a skewness value > 3 or kurtosis > 7, and there was no item with a floor or ceiling effect of > 75%. No item was deleted because of psychometric sensitivity.

#### Validity

Construct validity was assessed by EFA. The KMO value of 0.957 and significant Bartlett’s test met the requirements for factor analysis. In the EFA, six factors with 45 items were determined by eigenvalues (> 1), accounting for 58.60% of the total variance (Table 3).

**Table 3:** Eigenvalue and variance contribution rate of each factor

	Extraction sums of squared loadings			Rotation sums of squared loadings		
Factor	Total	% of variance	Cumulative %	Total	% of variance	Cumulative %
1	15.372	32.025	32.025	7.741	16.126	16.126
2	4.902	10.212	42.237	5.455	11.365	27.491
3	3.299	6.874	49.111	5.441	11.336	38.828
4	2.170	4.521	53.632	3.148	6.559	45.387
5	1.371	2.857	56.489	3.083	6.422	51.809
6	1.013	2.110	58.599	2.681	5.585	57.394
7	.790	1.646	60.245	1.296	2.701	60.094
8	.585	1.220	61.464	.658	1.370	61.464

Extraction method: Principle Axis Factoring

Hence, a six-factor solution was obtained. Factor 1 was named “Care integration” (14 items, 32.03% of variable), factor 2 “person- & community-centeredness” (9items, 10.21% of variable), factor 3 “Cultural competence” (8 items, 6.87% of variable), factor 4 “Professional integration” (5 items, 4.52% of variable), factor 5 “Organizational integration” (4 items, 2.85% of variable), factor 6 “Technical competence” (5 items, 2.11% of variable). Items 33,34, and 43 with poor factor loading (< 0.4) were removed from the questionnaires.

All subscales of the C-RMIC-MT provider version were positively and significantly correlated with each other, see Table 4. In addition, Pearson’s correlations between the C-RMIC-MT care provider version total score and two overall perceived coordination questions showed good construct validity. The correlation coefficient between total score and the question “How do you rate coordination among CCPs inside your CHS?” was 0.531(*p* < 0.01), while that between total score and the question “How do you rate coordination among CCPs outside your CHS?” was 0.554(p < 0.01). In addition, care providers who indicated a better care coordination ability were more satisfied about the adaptive reserve (r = 0.69, p < 0.01) .

**Table 4:** Correlation between subscale scores C-RMIC-MT provider version (n = 1610)

Variables	1	2	3	4	5	6
1. Person- & community- centeredness (item 1–9)						
2. Care integration (item 10–23)	.730^a^					
3. Professional integration (item 24–28)	.355^a^	.440^a^				
4. Organizational integration (item 29–32)	.118^a^	.120^a^	.156^a^			
5. Technical competence (item 33–37)	.164^a^	.320^a^	.422^a^	.026^a^		
6. Cultural competence (item 38–45)	.371^a^	.462^a^	.675^a^	.111^a^	.443^a^	

^a^ Correlation is significant at the 0.01 level (2-tailed)

#### Internal consistency reliability

The internal consistency analysis showed that the six dimensions of the C-RMIC-MT provider version could be reliably measured. Correlation coefficients between/among all items in the scale were > 0.4. Items scale correlations showed that all individual items highly correlated with their respective subscale compared with the competing scales. The C-RMIC-MT provider version is also a reliable instrument (Cronbach’s alpha 0.940) of 45 items to measure integrated care.

#### Goodness to fit factor model

A structural equation model with maximum likelihood evaluated he proposal model fit with RMSEA 0.056, SRMR 0.061, CFI 0.857, TLI 0.848. The model passed the majority of goodness-to-fit test by confirmation factor analysis.

## Discussion

Changing the behaviors of health professions is an essential precondition for achieving integrated care. Given this, it is essential for researchers and policy-makers to measure and evaluate the implementation of integrated care from the perspective of care providers. This study provides the first assessment of the validity and reliability of the C-RMIC-MT provider version. It was used to measure integrated primary care from the perspective of CCPs.

Statistical analysis indicated that the construct validity and internal consistency for C-RMIC-MT provider version (45 items, 6 subscale) were good. The proposed model also passed the majority of goodness-to-fit test. This suggests that the C-RMIC-MT provider version is a valuable psychometric tool for evaluating care integration perceived by care providers.

The C-RMIC-MT provider version yielded six dimensions reflecting parts of hypothesized dimensions of the RMIC (person- & community- centeredness, care integration, professional integration, organizational integration, technical competence, and cultural competence). The factor analysis leads us to conclude that care provider in CHSs of China don’t differentiate original clinical, organizational and system integration dimensions of integrated care. Care providers in previous study also found it difficult to differentiate between the organizational and system dimensions of RMIC [43]. The items hypothesized to belong to Person-centeredness dimension were absorbed by the community-centeredness dimension, which might reflect that care providers in CHSs of Chinese health systems don’t recognize it and differentiate between person-centeredness and community-centeredness.

The validation of RMIC-MT provider version in China primary care setting show different results comparing with its validation in 19 countries [25], which reflects cultural backgrounds. Most of the variance of C-RMIC-MT provider version was explained by care integration, while most of the variance in international validation was explained by cultural competence. It also highlights the importance of person and community-orientation (10.21% of variable) as a principle of integrated care, which was highlighted by the validation in 19 countries.

### Strengths of the tool and limitations of the study

This study reports the first validation of the C-RMIC-MT care provider version. In summary, this instrument has three highlights. First, based on the Rainbow Model, it is not only easy to use but also focuses on comprehensive dimensions comparing with previous international integrated care instruments. A recent systematic review found that none of the instruments for measuring integrated care assessed system or normative integration [11]. The C-RMIC-MT care provider version filled this measurement gap. Second, it measures integrated care from the perspective of care providers, different from traditional patient’s perspective. Third, as the first Chinese instrument to measure integrated care, it shows good psychometrics properties in Chinese primary care settings. It may be useful as a tool to monitor the degree of integrated care in a region over time, providing evidence of strategy adjustment.

However, the study had some limitations. First, while the number of respondents met the requirement (over 10 times the item number) and the response rate was very high, but only stakeholders from the public CHSs in a single district were presented. As mentioned, high homogeneity of the sampled CHSs might influence the validity assessment. Future studies with diverse samples crossing regions would be needed to further test the psychometric properties for the Chinese primary care context. Second, this was a computer-based assessment. Evidence suggests that a model of administration (such as computer-based vs pencil version) has little impact on psychometric properties of measures [44, 45]. But authors do lose the chance to explore reasons for the answers of respondents when the data are collected by computers rather than collected by paper questionnaire face-to-face. Therefore, we conducted six interviews with CCPs in six CHSs after data collection by computer, including two GPs, two nurses, a public health physician, and an internist, to find the influencing factors of professionals’ experiences with integrated care and potential effective collaboration mechanisms. It is necessary to have more communication with CCPs in further psychometric assessments of the C-RMIC-MT care provider version.

## Conclusions

Value-based integrated health systems with strong primary care are being built in the whole of China. Primary integrated care has the potential to achieve the triple aim, but implementation determines the final practical results. The Chinese RMIC-MT care provider version initially validated in this study exhibits good psychometric properties in Chinese primary context. Its application in China could contribute to the measurement of different dimensions of integrated primary care from the perspective of health professions, revealing problems during the implementation of related reforms, and then promoting the building of high-performing heath systems. Moreover, its validation and application in European countries may help in establishing international compassion for integrated primary care.

## Data Availability

The datasets used and analyzed during the current study are available from the corresponding author on reasonable request.

## Abbreviations

WHO: World Health Organization
RMIC: Rainbow Model of Integrated Care
RMIC-MT: Rainbow Model of Integrated Care-Measurement Tool
HEP: Health Economics and Policy
EV: English version
CV: Chinese version
CCP: Community care professional
CHS: Community health stations
GP: General practitioner
KMO: Kaiser-Meyer-Olkin
EFA: Exploratory factor analysis
SRMR: Standardized root-mean-square residual
CFI: Comparative fit index
TLI: Tucker-Lewis index
